# MTA2 promotes gastric cancer cells invasion and is transcriptionally regulated by Sp1

**DOI:** 10.1186/1476-4598-12-102

**Published:** 2013-09-08

**Authors:** Chenfei Zhou, Jun Ji, Qu Cai, Min Shi, Xuehua Chen, Yingyan Yu, Bingya Liu, Zhenggang Zhu, Jun Zhang

**Affiliations:** 1 Department of Surgery, Shanghai Institute of Digestive Surgery, Ruijin Hospital, Shanghai Jiao Tong University School of Medicine, No. 197 Ruijin er Road, Shanghai 200025, P.R. China; 2 Department of Clinical Oncology, Ruijin Hospital, Shanghai Jiao Tong University School of Medicine, No. 197 Ruijin er Road, Shanghai 200025, P.R. China

**Keywords:** Gastric cancer, MTA2, Cell invasion, Transcriptional regulation, Sp1

## Abstract

**Background:**

MTA2 gene belongs to metastasis associated family, and is highly expressed in some solid tumors, including gastric cancer. Its biological function in gastric cancer is currently undefined.

**Methods:**

Metastasis-associated tumor gene family 2 (MTA2) and transcription factor specificity protein 1 (Sp1) expression were detected in 127 gastric cancer samples by immunohistochemistry staining. SGC-7901 and AGS gastric cancer cell lines transfected by MTA2 shRNA was used for biological function investigation. Binding and regulation activities of Sp1 on MTA2 promoter were investigated by chromatin immunoprecipitation and luciferase reporter gene.

**Results:**

The expression rate of MTA2 in gastric cancer tissues was 55.9% (71/127), and its expression was closely related to the depth of tumor invasion, lymph nodes metastasis, and TNM staging. MTA2 knockdown in human SGC-7901 and AGS gastric cancer cells significantly inhibited migration and invasion *in vitro*, and disrupted structure of cytoskeleton. MTA2 knockdown also attenuated xenografts growth and lung metastasis in nude mice model. MTA2 expression was positively correlated with transcription factor Sp1 in gastric cancer tissues (*r* = 0.326, *P* < 0.001). Sp1 bound to human MTA2 gene promoter at region from -1043 bp to -843 bp. Transcriptional activity of MTA2 promoter could be enhanced by Sp1 overexpression.

**Conclusions:**

MTA2 knockdown impairs invasion and metastasis of gastric cancer cells, and attenuates xenografts growth *in vivo*. Sp1 regulates MTA2 expression at transcriptional level.

## Background

Gastric cancer is one of the major malignancies in mainland of China, with high incidence and mortality [1]. The newly diagnosed gastric cancer patients in China account for 40% worldwide cases every year [2,3]. Furthermore, over 80% gastric cancer patients in this developing country are diagnosed at advanced stage, resulting in a relative poor outcome [4,5].

Distant metastasis is the major cause of death for cancer patients. Initiation of metastasis consists of multiple steps and involves abundant molecules events [6,7]. Mechanisms of metastasis in gastric cancer are still under investigation. Metastasis-associated tumor gene family (MTA) has three members, MTA1, MTA2 and MTA3. MTA1 overexpression was observed in some solid tumors, including breast, esophageal, pancreatic, hepatocellular carcinoma [8], and correlated with cancer cell invasion and metastasis [9,10]. MTA2 has high homology with MTA1 in protein alignment, and also consists in nucleosome remodeling and histone deacetylase (NuDR) complex [11,12]. These results suggested that function of MTA2 might be similar to MTA1. Currently, its biological function in gastric cancer is still unclear.

The regulation of MTA family has not been fully elucidated. It was reported that mouse Mta2 gene was regulated by transcription factor specificity protein 1 (Sp1) [13]. Although amino acid sequences of MTA2 between human and mouse are highly identical [11], the promoter sequences are different. Sp1 was found to be overexpressed in gastric cancer tissues, and had positive correlation with MTA2 [14-16]. Those results hinted potential regulatory role of Sp1 in MTA2 transcription.

In present study, the biological function of MTA2 protein in gastric cancer was assessed both *in vitro* and *in vivo*, the role of Sp1 in transcriptional regulation of human MTA2 gene promoter was also investigated.

## Results

### MTA2 protein was highly expressed in gastric cancer tissues

**Figure 1:**
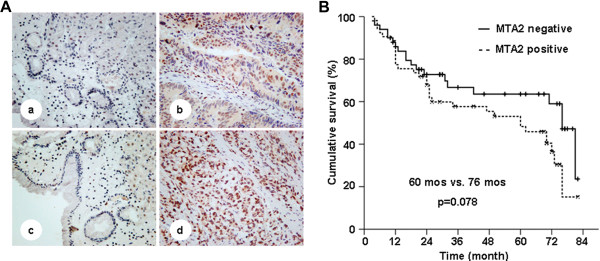
**MTA2 in paired mucosa beside tumor and tumor tissues, and survival curve of patients. A**, MTA2 expression in paired gastric mucosa beside tumor and tumor tissues from two patients (a, b and c, d) were detected by IHC (×100). **B**, Patients with MTA2 positive expression had a shorter median overall survival than those with MTA2 negative (60 mos vs. 76 mos, p = 0.078).

The results of immunohistochemistry staining revealed that, the positive rate of MTA2 protein in gastric cancer tissues was 55.9% (71/127). MTA2 protein was mainly localized in the nuclear of gastric cancer cells, and its expression was much more dominant in tumor tissues than that in neighboring gastric mucosa (Figure 1A). MTA2 protein expression was closely related with depth of tumor invasion (*P* = 0.003), lymph nodes metastasis (*P* = 0.019), and TNM staging (*P* = 0.007), whereas no correlation with Lauren’s classification and tumor differentiation (Table 1). Patients with MTA2 expression had a shorter overall survival time with marginally significance (60 mos vs. 76 mos, *P* = 0.078, Figure 1B). By multivariate analysis, TNM staging was an independent prognostic factor (*P* < 0.001, HR 2.165, 95% CI 1.612 to 2.909). However, no survival difference was observed in patients with different MTA2 status in identical TNM staging (Additional file 1: Figure S1).

**Table 1:** Correlation between MTA2 and clinicopathological characteristics of gastric cancer patients

**Clinicopathological characteristics**		**MTA2**		***P***
		**-**	**+**	
Tumor invasion				
	T1	10	3	0.003
	T2	15	8	
	T3	25	50	
	T4	6	10	
Lymph node metastasis				
	Negative	28	21	0.019
	Positive	28	50	
TNM staging				
	I	20	9	0.007
	II	13	14	
	III	13	32	
	IV	10	16	
Lauren’s type				
	Intestinal	28	31	>0.05
	Diffuse	28	40	
Differentiation				
	High	16	15	>0.05
	Low	40	56	

### MTA2 knockdown attenuated migration and invasion of gastric cancer cells

**Figure 2:**
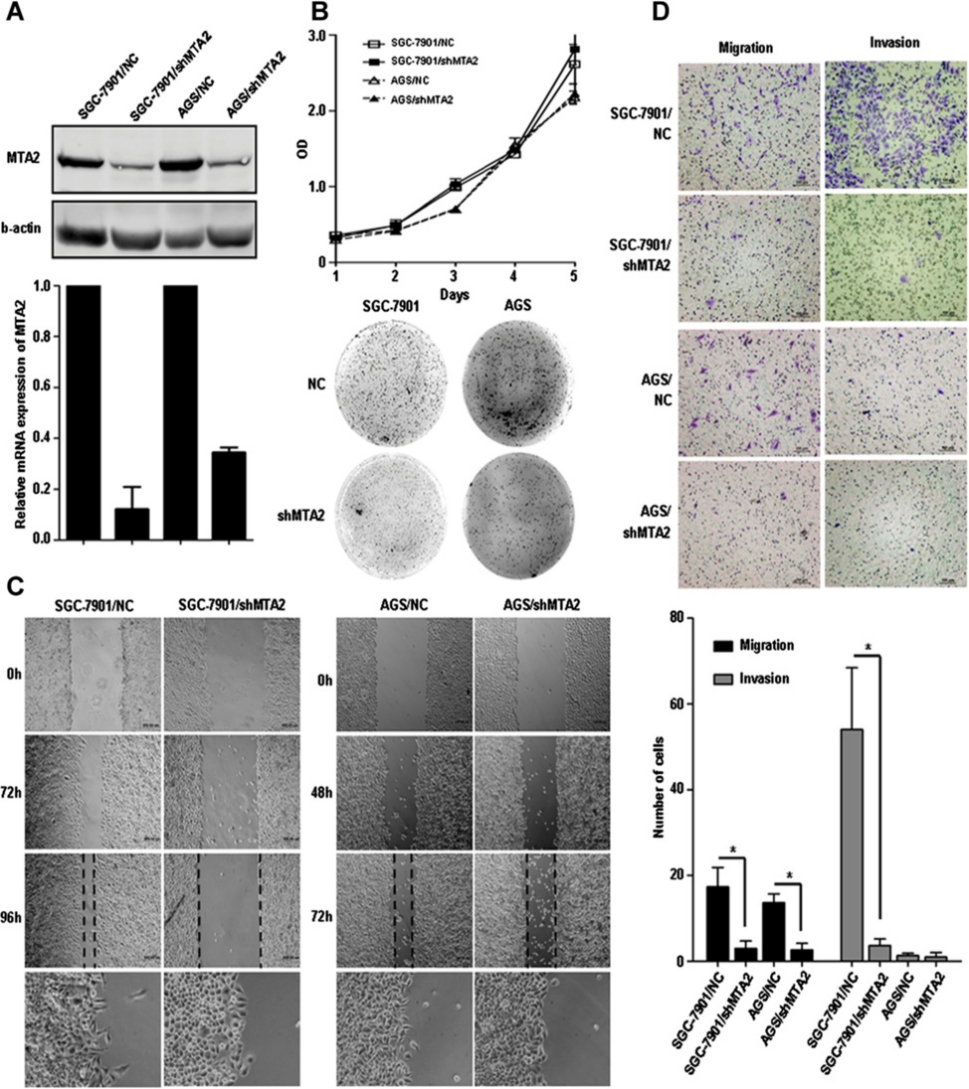
**MTA2 knockdown inhibited cell migration and invasion *in vitro*. A**, Protein and mRNA expression of MTA2 were detected in shMTA2 stable transfected cells and negative control (NC). **B**, *In vitro* cell growth were analyzed by proliferation assay and colony formation assay. **C**, Cell mobility was investigated by wound healing assay. **D**, Capabilities of migration and invasion were analyzed by Transwell assay (Migration: without Matrigel; Invasion: coated by Matrigel).

MTA2 expression in gastric cancer cell lines SGC-7901 and AGS was knocked down by shRNA transfection, and cells transfected by vector was identified as negative control (Figure 2A). In proliferation assay, no significant difference was observed between MTA2 knockdown group and control group. In colony formation assay, the number of clones was similar between two groups, but the size of clones formed in MTA2 knockdown cells was smaller than those in control group (Figure 2B).

The wound healing course of MTA2 knockdown group was significantly slower than that in control group. Furthermore, at the edge of scratch, pseudopodia of control cells could extend perpendicular to the free space of scratch, which was found in MTA2 knockdown group (Figure 2C). For SGC-7901 cells, the numbers of cells penetrating the membrane of chambers in both migration and invasion assays were significantly less in MTA2 knockdown group than those in control group (Migration: 3.0 ± 1.7 vs. 17.3 ± 4.5, *P* = 0.007; Invasion: 3.7 ± 1.5 vs. 54.0 ± 14.4, *P* = 0.004, Figure 2D), respectively. For AGS cell line, the result of migration assay was similar (2.7 ± 1.5 vs. 13.7 ± 2.1, *P* = 0.002, Figure 2D), and in invasion assay, number of cells were low in both groups. MTA2 expression in AGS/shMTA2 cells was rescued by transient transfection of MTA2 plasmid. Migration ability of AGS/shMTA2 cells was partially recovered (Additional file 1: Figure S2).

### MTA2 knockdown disturbed the structure of actin cytoskeleton

**Figure 3:**
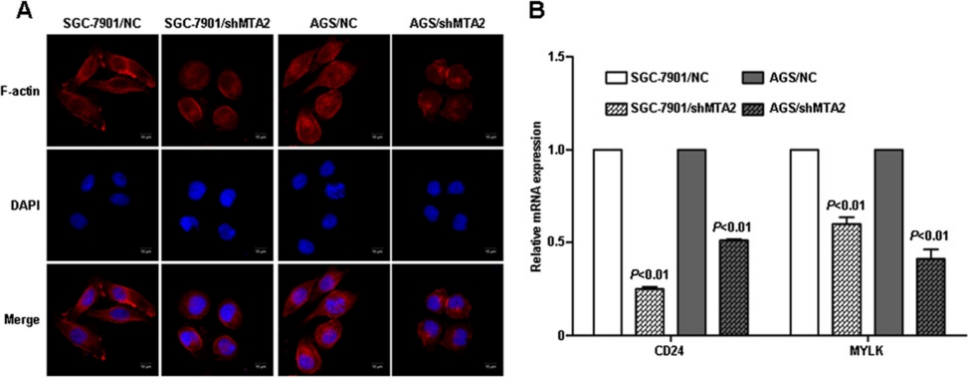
**MTA2 knockdown disturbed the structure of actin cytoskeleton. A**, F-actin cytoskeleton structure was visualized by immunofluorescence staining. **B**, Relative mRNA expression of CD24 and MYLK was detected by qRT-PCR.

In order to confirm the morphological changes observed in wound healing assay, the structure of actin cytoskeleton was observed. In MTA2 knockdown cells, the structure of fibrous actin (F-actin) was less than cells in control group. Cell morphology was changed from spindle to spherical after MTA2 knockdown (Figure 3A). The expression of CD24 and MYLK, genes related with cytoskeleton regulation were assessed by qRT-PCR. These two genes were found to be significantly reduced in MTA2 knockdown cells than those in control group (Figure 3B).

### MTA2 knockdown attenuated xenograft growth and lung metastasis

**Figure 4:**
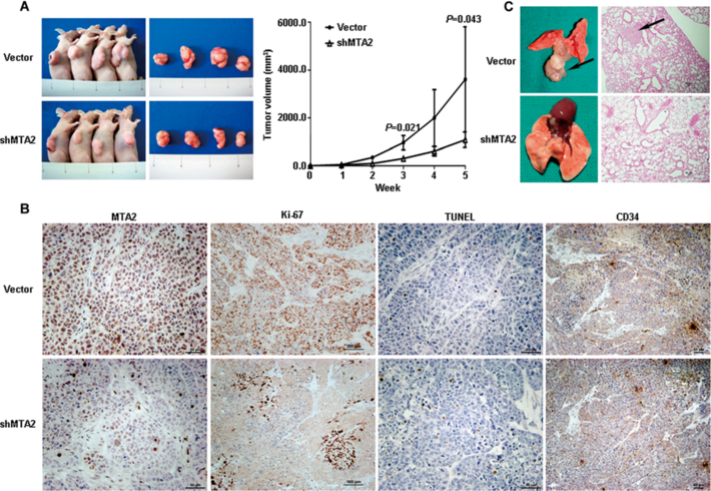
**MTA2 knockdown reduced xenografts growth and lung metastasis of SGC-7901 cells. A**, Xenografts of SGC-7901/NC and SGC-7901/shMTA2 cells were measured every 7 days for four weeks. **B**, Expression of MTA2, Ki-67 and CD34 was detected by immunohistochemistry. Cell apoptosis was assessed by TUNEL assay. **C**, Lung metastasis of SGC-7901/Vector cells was detected by H.E. staining.

The growth rate of MTA2 knockdown SGC-7901 xenografts was slower than that in control group (*P* = 0.043, Figure 4A), and the average tumor weight of xenografts was also lower (0.82 ± 0.08 g vs. 2.12 ± 0.58 g, *P* = 0.021). MTA2 expression in xenografts was detected both by western blot (Additional file 1: Figure S3) and IHC (Figure 4B). Ki-67 expression was significantly reduced in MTA2 knockdown group, while apoptosis index (3.3 ± 0.6% vs. 3.0 ± 1.0%, *P* = 0.643) and microvessel density were similar in both groups (Figure 4B). In lung metastasis models, four mice in control group (4/9) developed pulmonary metastase (Figure 4C). Per contra, no mice were found to develop lung metastasis in MTA2 knockdown group (0/9).

### MTA2 in gastric cancer tissues was related with Sp1 expression

**Figure 5:**
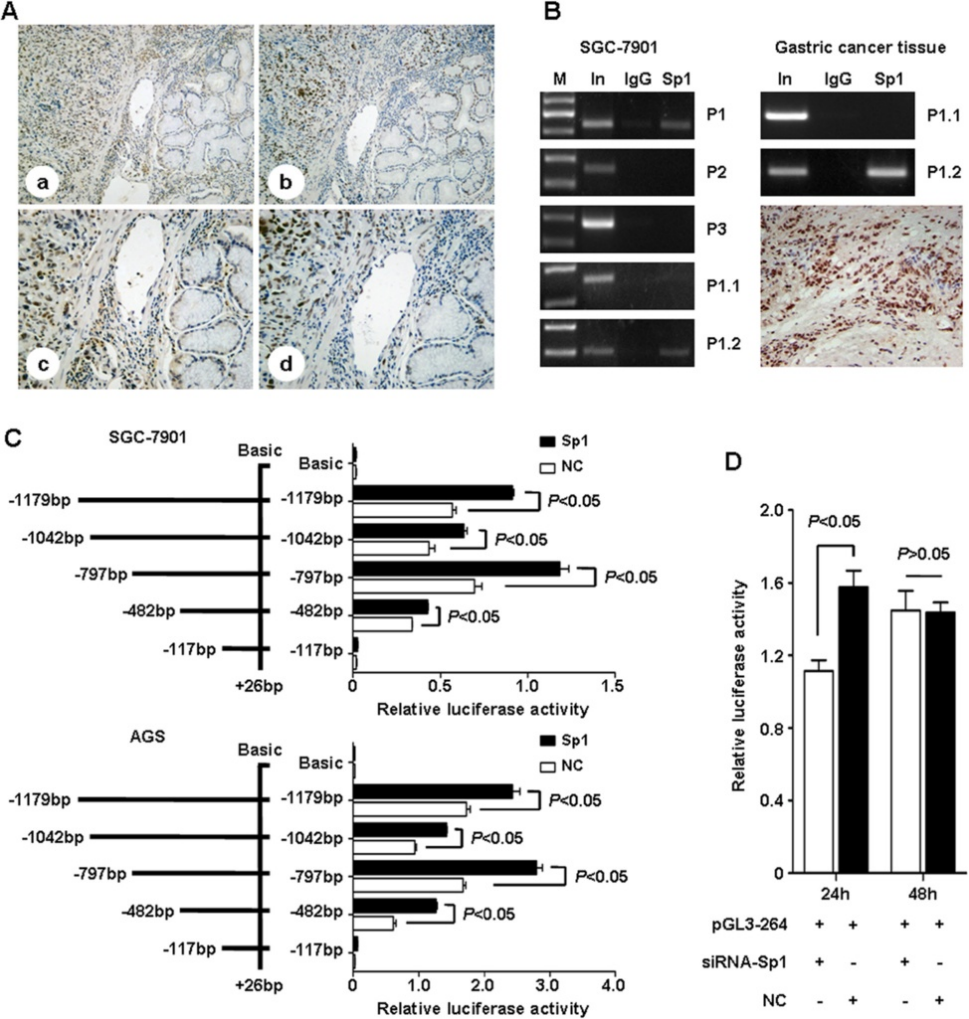
**Sp1 enhance transcriptional activity of human MTA2 gene promoter. A**, Expression of MTA2 (a × 100, c × 200) and Sp1 (b × 100, d × 200) in gastric cancer tissues were detected by IHC. **B**, DNA fragment pulled down with Sp1 antibody from SGC-7901 cells was amplified by PCR using P1 (-1179 bp ~ -843 bp), P2 (-948 bp ~ -671 bp), P3 (-559 bp ~ -301 bp), P1.1 (-1179 bp ~ -1023 bp) and P1.2 (-1043 bp ~ -843 bp). P1.1 (-1179 bp ~ -1023 bp) and P1.2 (-1043 bp ~ -843 bp) were used for gastric cancer tissue with Sp1 high expression. **C**, Luciferase reporters containing five different length of MTA2 promoter were co-transfected with Sp1 plasmid into SGC-7901 and AGS cells. Relative luciferase activity was detected, respectively. **D**, pGL3-264 luciferase reporter was co-transfected with siRNA-Sp1 into SGC-7901 cells, and relative luciferase activity was detected. M: Marker; In: Input; P: Primer; NC: negative control.

Both MTA2 and Sp1 protein had similar expression pattern in gastric cancer tissues (Figure 5A). The expression rate of MTA2 was significant higher in those concomitantly with Sp1 expression (Table 2, *P* < 0.01). Positive correlation was found between the expression of these two proteins (*r* = 0.326, *P* < 0.001). Concomitant expression of MTA2 and Sp1 was also observed in gastric cancer cell lines (Additional file 1: Figure S4).

**Table 2:** Expression of Sp1 and MTA2 in gastric cancer tissues

		**MTA2**		**P**
		**-**	**+**	
Sp1	-	35	20	<0.01
	+	21	51	

### Binding area of Sp1 on MTA2 promoter located at region from -1043 bp to -843 bp

The results of ChIP assay in SGC-7901 cells demonstrated that, PCR product could be amplified by primer1 (-1179 bp to -843 bp) and primer1.2 (-1043 bp to -843 bp), rather than other 3 pairs. To confirm the *in vitro* results, ChIP assay was also performed using gastric cancer tissues with Sp1 high expression. PCR product could also be amplified by primer1.2 rather than primer1.1 (Figure 5B).

### Sp1 overexpression enhanced the transcriptional activity of MTA2 promoter

Reporter plasmids were co-transfected into SGC-7901 cells with either Sp1 plasmid or vector. The activities of pGL3-1179, pGL3-1042, pGL3-797 and pGL3-482 were significantly elevated by Sp1 introduction, whereas the activity of pGL3-117 was extremely low, and had no response to Sp1 introduction (Figure 5C). Similar results were also demonstrated in AGS cells (Figure 5C). MTA2 mRNA expression was increased after Sp1 overexpression in MKN45 cells, whereas the protein level was not significantly changed (Additional file 1: Figure S5).

To verify the transcriptional role of Sp1 in MTA2 promoter, Sp1 specific siRNA was co-transfected into SGC-7901 cells with pGL3-264, the reporter plasmid containing Sp1 binding area. Luciferase activity was significantly reduced by siRNA transfection after 24 h (Figure 5D).

## Discussion

MTA2 is an essential part for NuRD complex and activity [17]. The pathological and biological characteristics of MTA2 in malignancies were rarely reported. MTA2 overexpression was first described in cervical cancer [11]. Hereafter, its aberrant expression was found in ovarian epithelial cancer, hepatocellular cancer, non-small cell lung cancer, and correlated with invasive phenotypes such as large tumor size, poor differentiation and lymph node metastasis [18-20].

The results of this study validated the patterns of MTA2 expression in gastric cancer tissues as we previously reported [16]. Whether MTA2 could be served as a prognostic factor of gastric cancer was still unclear [20]. Patients with MTA2 expression had a trend of worse outcome. However, survival analysis stratified by TNM staging showed no difference between MTA2 positive or negative groups (Additional file 1: Table S1). Therefore, even MTA2 expression was found to be related with TNM staging, it is still couldn’t be considered an independent prognostic factor.

The process of cancer invasion and metastasis is complicated, tumor cells break through the barrier of normal tissue, invade into surrounding extracellular matrix and circulation, then extravasate into distant organs to form metastasis foci. Cell mobility and invasion ability play important roles in these processes [7]. The results of present study revealed the role of MTA2 in regulating gastric cancer cell mobility and invasion. Fu et al. also found that invasion and metastasis abilities of mouse 4 T1 mammary tumor cells were reduced after Mta2 gene interference [21]. These results indicated that MTA2 might participate in the process of tumor progression.

The mechanisms of MTA2 in regulating cancer cell mobility and invasion were unclear. MTA2 was reported to serve as a component of Twist complex, and participated in inhibition of E-cadherin gene transcription. In this study, we found that knockdown of MTA2 gene expression could alter gastric cancer cell morphology and disturb F-actin structure. Furthermore, reduced mRNA expression of both CD24 and MYLK were found in MTA2 knockdown cells. Cytoskeleton remolding is essential for controlling cell motility and cancer invasion [22]. Up-regulation of CD24 could increase actin cytoskeletal remolding dynamic and enhance contractile forces to facilitate cancer cell invasion [23,24]. Myosin light chains phosphorylated by MYLK increase its interaction with actin filaments, and promote cell protrusion and contraction [25]. NuRD complex, which MTA2 consists in, regulates gene expression by remolding the structure of nucleosome. Alteration of CD24 and MYLK expression might be one of the mechanisms for MTA2 to modulate cytoskeleton and participate in cell invasion.

The impacts of MTA2 knockdown on gastric cancer cell growth *in vitro* seemed to be a paradox. MTA2 knockdown didn’t interfere gastric cancer cell growth in proliferation assay, but colony formation was attenuated. This divergence might be attributed to different culture conditions. Cells adhered to culture plates in proliferation assay, whereas was suspended in agar during colony formation. Adhesion to substratum is necessary for normal adhering cells survival and growth [26]. Tumor cells, however, can survive and grow in adhesion absent or anchorage-independent condition, which is important for tumorigenesis as well as metastasis [27]. Colony formation assay is a convenient assay to assess the capability of cell growth in anchorage-independent condition, and is closely related to the *in vivo* situation [28]. Attenuation of tumorigenesis and metastasis *in vivo* validated the result of colony formation assay, indicated that MTA2 knockdown could impair the anchorage-independent growth of gastric cancer cells. Exogenous expression of MTA2 in SaoS-2 cells could enhance cell growth in colony formation assay. Reducing of p53 acetylation and expression after MTA2 overexpression suggested that p53 inhibition might be one of the mechanisms of MTA2 regulating anchorage- independent growth [29]. Increased p53 expression was also found in MTA2 knockdown cells in this study (Additional file 1: Figure S6).

Transcription factor Sp1 plays an important role in physiological process and also in human cancer progression by regulating transcription of diverse downstream genes [30,31]. Concomitant expression of MTA2 and Sp1, and high GC-content sequence in proximal region of human MTA2 promoter, the potential Sp1 binding region [32], indicated that MTA2 might also be a downstream gene of Sp1 in gastric cancer. The results of ChIP and luciferase activity assays demonstrated that Sp1 could bind to MTA2 promoter, and enhance its transcriptional activity.

In ChIP assay, we found that the binding area of Sp1 on MTA2 promoter was at -1043 bp to -843 bp. However, promoter downstream of this fragment could also be regulated by Sp1 introduction. The characteristics of Sp1 might attribute to this phenomenon. As a critical transcription factor, Sp1 can regulate the expression of abundant genes, including many transcription factors, and even itself [33]. Overexpression of Sp1 might trigger the activities of some transcription factors, and increased the transcriptional activity of MTA2 promoter without direct binding.

On the other hand, multiple transcription factors typically bind together and form a transcriptional complex [34]. Alteration of one factor might not abrogate the function of the complex. Luciferase activity recovered at 48 h after Sp1 knockdown suggested a compensation mechanism might exist. Xia et al. reported that cooperation of transcription factor ETS or existence of Sp3 might contribute to stable Mta2 protein level in mouse ES cells after Sp1 knockdown [13]. In our study, several transcription factors’ binding sites were predicted in fragment from -1042 bp to -797 bp, including AP-1, NF-1 and E2F which were reported had interaction with Sp1 [30]. Although the regulatory function of Sp1 on MTA2 promoter was demonstrated in this study, the in-detailed mechanisms of MTA2 transcription need to be further clarified.

In conclusion, aberrant expression of MTA2 in gastric cancer cells participates in cell mobility and invasion. Overexpression of Sp1 could regulate the transcriptional activity of MTA2 promoter and might partially contribute to MTA2 expression in gastric cancer.

## Materials and methods

### Ethical statement

Written informed consent was obtained from all participants, and study protocol was approved by the ethics committee of Ruijin Hospital, Shanghai Jiaotong University School of Medicine. All mouse experiments were approved (Permit#20110930) by the Animal Care and Use Committee and conducted an accord with the Guide for the Care and Use Laboratory Animals of Ruijin Hospital, Shanghai Jiaotong University School of Medicine.

### Patients and samples

Paraffin-embedded specimens from 127 gastric cancer patients underwent standard D2 resection, were collected from Department of Surgery, Shanghai Ruijin Hospital. All samples were pathologically confirmed as gastric adenocarcinoma. Clinicopathological data were reviewed, and were listed in Additional file 1: Table S1. TNM staging classification was based on criteria of American Joint Committee on Cancer (AJCC, 6th edition). Overall survival was recorded from 103 patients.

### Cell lines and transfection

Gastric cancer cell lines SGC-7901, AGS, MKN45, BGC-823 were purchased from Shanghai Institute of Biological Sciences, Chinese Academy of Sciences. Hs746T was purchased from ATCC. Cells were cultured by RPMI-1640 medium with 10% fetal calf serum at 37°C and 5% CO_2_. Five shRNA plasmids targeting different regions of MTA2 mRNA were purchased from Sigma. Plasmid shRNA-74 had the strongest efficiency, and its interference sequence was 5′-CCC TCT TGA ATG AGA CAG ATA CTC GAG TAT CTG TCT CAT TCA AGA GGG-3′. Stable transfected cells were selected by puromycin (5 μg/ml). Luciferase reporter plasmids constructed by pGL3-Basic vector (Promega) and truncated MTA2 promoters were co-transfected with Sp1 plasmid (persevered in our institute) into SGC-7901 and AGS cells. Relative luciferase activity was detected at 48 h after transfection by dual-luciferase assay system (Promega). Ratio of reporter plasmids and control plasmid (pRL-TK, Promega) was 20:1. The concentration of Sp1-siRNA (Invitrogen) transfected into SGC-7901 cells was 50 nM. Lipofectamine 2000 (Invitrogen) was used as transfection regent.

### Immunohistochemistry staining (IHC)

Immunohistochemistry staining was performed on 4 μm-thick slices following EnVision two-step procedure of Dako REAL™ Envision™ Detection System (Dako). Slides were incubated by primary antibodies including MTA2 (1:200, Santa Cruz), Sp1 (1:200, Santa Cruz), Ki-67 (1:50, Dako), and CD34 (1:150, Dako), respectively, followed incubated by HRP labeled secondary antibody and were visualized by diaminobenzidine.

Both MTA2 and Sp1 proteins were localized in cell nuclear stained as brownish granules. The expression status of MTA2 and Sp1 was determined by product score of average percentage and intensity of positive cells under 5 random high-power fields [14]. Score for percentage: <5% (0), 5%-25% (1), 25%-50% (2), 50%-75% (3), and >75% (4); for intensity: no staining (0), light brown (1), brown (2), and dark brown (3). For MTA2, score of 0 and ≥1 was defined as negative and positive, respectively. For Sp1, score of ≤3 and >3 was defined as negative and positive, respectively.

### Western blot

One hundred micrograms protein were fractionated by 10% SDS-PAGE gel and transferred to PVDF membranes. PVDF membranes were blocked by 5% skim milk for 1 h, then were incubated overnight at 4°C with primary antibodies, including MTA2 (1:1000, BETHYL), Sp1 (1:500, Santa cruz) and HDAC1 (1:1000, Cell signaling technology). Fluorescent secondary antibodies (1:15000, LI-COR) and infrared imaging system (LI-COR) were used to visualize the protein bands.

### Quantitative reverse transcription-polymerase chain reaction (qRT-PCR)

RNA was extracted by Trizol reagent method. Reverse transcription in 20 μl-system was preformed following protocol of Applied Biosystems. Primers for qRT-PCR were MTA2: F-TGT ACC GGG TGG GAG ATT AC, R-TGA GGC TAC TAG AAA TGT CCC TG; CD24: F-CTC CTA CCC ACG CAG ATT TAT TC, R-AGA GTG AGA CCA CGA AGA GAC; MYLK: F- CCC GAG GTT GTC TGG TTC AAA, R-GCA GGT GTA CTT GGC ATC GT. Quantitative mRNA expression was measured by ABI Prism 7900HT sequence detection system (Applied Biosystems), and relative mRNA expression was calculated by comparative Ct method.

### *In vitro* proliferation and colony formation assay

One thousand cells per well were plated and adhered in 96-well plates. Cell growth curves were assessed by Cell Counting Kit-8 (Dojindo) for 5 days. Dissolved agar was used in colony formation assay. Two milliliter of 0.4% ~ 0.6% agar was added into 6-well plate as bottom gel. One thousand cells suspended in 4 ml of 0.2% diluted agar were poured on bottom gel, and were cultured for 2 weeks. MTT was used to staining.

### Wound healing and transwell assay

Cells were plated in 12-well plates with concentration more than 90%. Wounds were scratched by pipette tips and photographed every 24 h. Suspended cells were washed by PBS buffer. Serum-free medium was used for cell culture.

In invasion assay, 100 μl of diluted Matrigel (BD Biosciences) was used to coat the chambers’ membrane (8 μm for 24-well plate, Millipore). Fifty thousand cells in 100 μl serum-free medium were added into the upper chambers, with full medium in lower chambers, then, cultured for 48 h. For migration assay, cells were cultured for 24 h under the same conditions without Matrigel. Cells were fixed by 10% formalin, and stained by 0.1% crystal violet, and were photographed and counted by inverted microscope.

### Immunofluorescence

Cells were seeded and cultured on glass slides. After 24 h, cells were fixed by 4% paraformaldehyde for 30 min, penetrated by 0.1% Triton X-100 for 15 min, and blocked by 5% BSA for 30 min. Then, cells was incubated with anti-actin antibody (1:80, Acti-stain™ 555 Fluorecent Phalloidin, Cytoskeleton) for 45 min, and mounted by VECTASHIELD Mounting Medium with DAPI (Vector Labs). The images were obtained by inverted ZEISS LSM710 confocal microscope (40× oil lens) (Carl Zeiss), with ZEN 2009 Light Edition software (Carl Zeiss).

### Subcutaneous nude mouse xenografts

Eight 4-week-old male BALB/c nude mice (Institute of Zoology, China Academy of Sciences) were randomly divided into 2 groups (4 for each group). One million SGC-7901/Vector or SGC-7901/shMTA2 cells in 100 μl PBS were inoculated subcutaneously. Tumor nodules were measured every 7 days after its length reached over 4 mm, and was calculated by following formula: V = (Width^2^ × Length)/2 [35]. Xenografts were collected at 5th week for immunohistochemistry staining and protein extraction.

### Pulmonary metastases models

Twenty 4-week-old male BALB/c nude mice (Institute of Zoology, China Academy of Sciences) were randomly divided into 2 groups (10 for each group). One million SGC-7901/Vector or SGC-7901/shMTA2 cells in 100 μl PBS were injected by tail vein, respectively. One mouse from each group was sacrificed at one month after injection to evaluate the metastasis formation. Two months later, all mice were sacrificed. Pulmonary metastases were checked by gross specimens and microscopy.

### Chromatin immunoprecipitation (ChIP)

The ChIP assay was performed following the protocol of chromatin immunoprecipitation kit (Millipore). Protein and DNA was crosslinked by formaldehyde, extracted by SDS lysis buffer, and sheared by sonication. Sp1 antibody (Santa Cruz) was used in immunoprecipitation. After purification of precipitated DNA, PCR was performed. The primers used for PCR were listed in Additional file 1: Table S2. PCR products were fractionated by gel electrophoresis and photographed.

### Luciferase reporter plasmid, site specific mutagenesis and promoter activity analysis

Primers for amplification of MTA2 promoter were listed in Additional file 1: Table S3, with Kpn I site at 5′ end of forward primer and Bgl II site at reverse primer. MTA2 promoter fragments were amplified from human genomic DNA, and were inserted into pGL3-Basic vector. Six reporter plasmids were constructed and named as pGL3-1179, pGL3-1042, pGL3-797, pGL3-482, pGL3-117 and pGL3-264. Single-tube luminometer (Promega) was used for dual-luciferase reporter assay (Promega) following the manual.

### Statistics

Chi-square test was used to analyze the categorical variables. Correlation between MTA2 and Sp1 expression was analyzed by Spearman test. Log-rank test in Kaplan-Meier method and Cox regression test was used to assess patients’ outcome and prognostic factors. Independent-samples *t* test was used in quantitive data analysis. *P*-value <0.05 was considered as statistical significant. All tests were preformed by SPSS 13.0 software (SPSS Inc.).

## Abbreviations

MTA2: Metastasis-associated tumor gene family 2; NuRD: Nucleosome remodeling and deacetylase; Sp1: Specificity protein 1; OS: Overall survival; IHC: Immunohistochemistry; AJCC: American joint committee on cancer; qRT-PCR: Quantitative reverse transcription-polymerase chain reaction; ChIP: Chromatin immunoprecipitation3.

## Competing interests

The authors declare that they have no competing interests.

## Authors’ contributions

Conceived and designed the experiments: CZ, JZ, BL, YY and ZZ. Performed the experiments: JJ, MS, QC and XC. Analyzed the data: CZ, JJ and JZ. Wrote the manuscript: CZ and JZ. All authors read and approved the final manuscript.

## Supplementary Material

Additional file 1: Figure S1 Survival curve of Patients. Patients have no survival difference with different MTA2 status in identical TNM staging. **Figure S2.** MTA2 expression and migration ability of AGS/shMTA2 cells. MTA2 expression was down-regulated in AGS/shMTA2 cells and was rescued by transient transfection of MTA2 plasmid. Migration ability of AGS/shMTA2 cells was partially recovered. **Figure S3.** MTA2 expression in xenografts was detected by western blot. MTA2 expression was down-regulated in xenografts of SGC-7901/shMTA2 cells. **Figure S4.** Expression of MTA2 and Sp1 in gastric cancer cell lines. Concomitant expression of MTA2 and Sp1 was observed in gastric cancer cell lines. **Figure S5.** MTA2 mRNA and protein expressions in Sp1 over-expressed MKN45 cells. MTA2 mRNA expression was increased after Sp1 overexpression in MKN45 cells, whereas the protein level was not significantly changed. **Figure S6.** p53 expression in MTA2 knockdown cells. p53 expression was increased in SGC-7901/shMTA2 and AGS/shMTA2 cells. **Table S1.** Clinicopathological characteristics of 127 gastric cancer patients. **Table S2.** Primers for PCR in chromatin immunoprecipitation. **Table S3.** Primers for construction of luciferase reporter plasmid. Click here for file
