# Supplementary material for: MTA2 promotes gastric cancer cells invasion and is transcriptionally regulated by Sp1
Source: Mol Cancer. 2013 Sep 8;12:102. doi: 10.1186/1476-4598-12-102 (PMC3851872; doi:10.1186/1476-4598-12-102)
Supplement: Additional file 1: Figure S1 — Survival curve of Patients. Patients have no survival difference with different MTA2 status in identical TNM staging. Figure S2. MTA2 expression and migration ability of AGS/shMTA2 cells. MTA2 expression was down-regulated in AGS/shMTA2 cells and was rescued by transient transfection of MTA2 plasmid. Migration ability of AGS/shMTA2 cells was partially recovered. Figure S3. MTA2 expression in xenografts was detected by western blot. MTA2 expression was down-regulated in xenografts of SGC-7901/shMTA2 cells. Figure S4. Expression of MTA2 and Sp1 in gastric cancer cell lines. Concomitant expression of MTA2 and Sp1 was observed in gastric cancer cell lines. Figure S5. MTA2 mRNA and protein expressions in Sp1 over-expressed MKN45 cells. MTA2 mRNA expression was increased after Sp1 overexpression in MKN45 cells, whereas the protein level was not significantly changed. Figure S6. p53 expression in MTA2 knockdown cells. p53 expression was increased in SGC-7901/shMTA2 and AGS/shMTA2 cells. Table S1. Clinicopathological characteristics of 127 gastric cancer patients. Table S2. Primers for PCR in chromatin immunoprecipitation. Table S3. Primers for construction of luciferase reporter plasmid. [file 1476-4598-12-102-S1.doc]

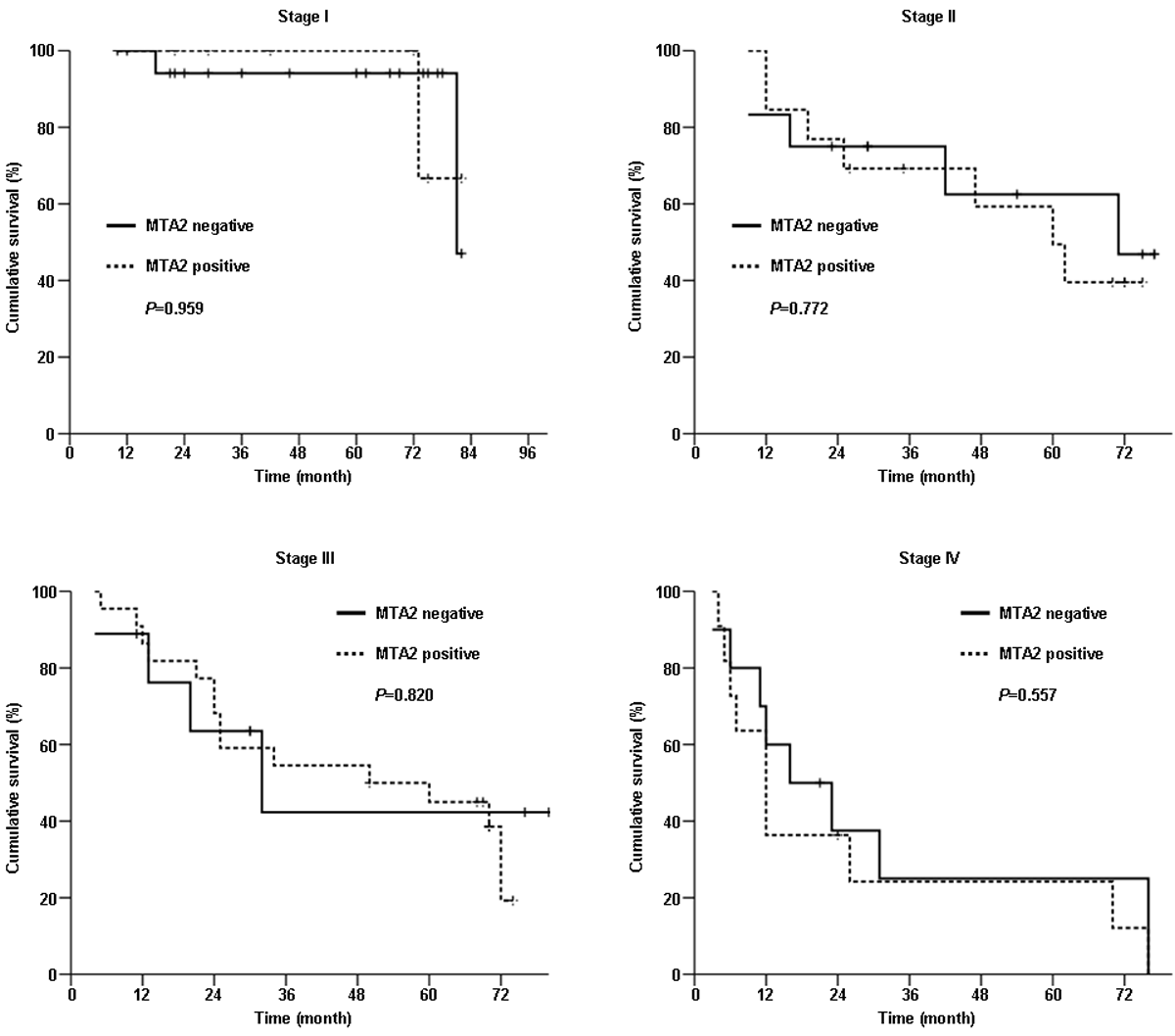


Supplementary Figure S1


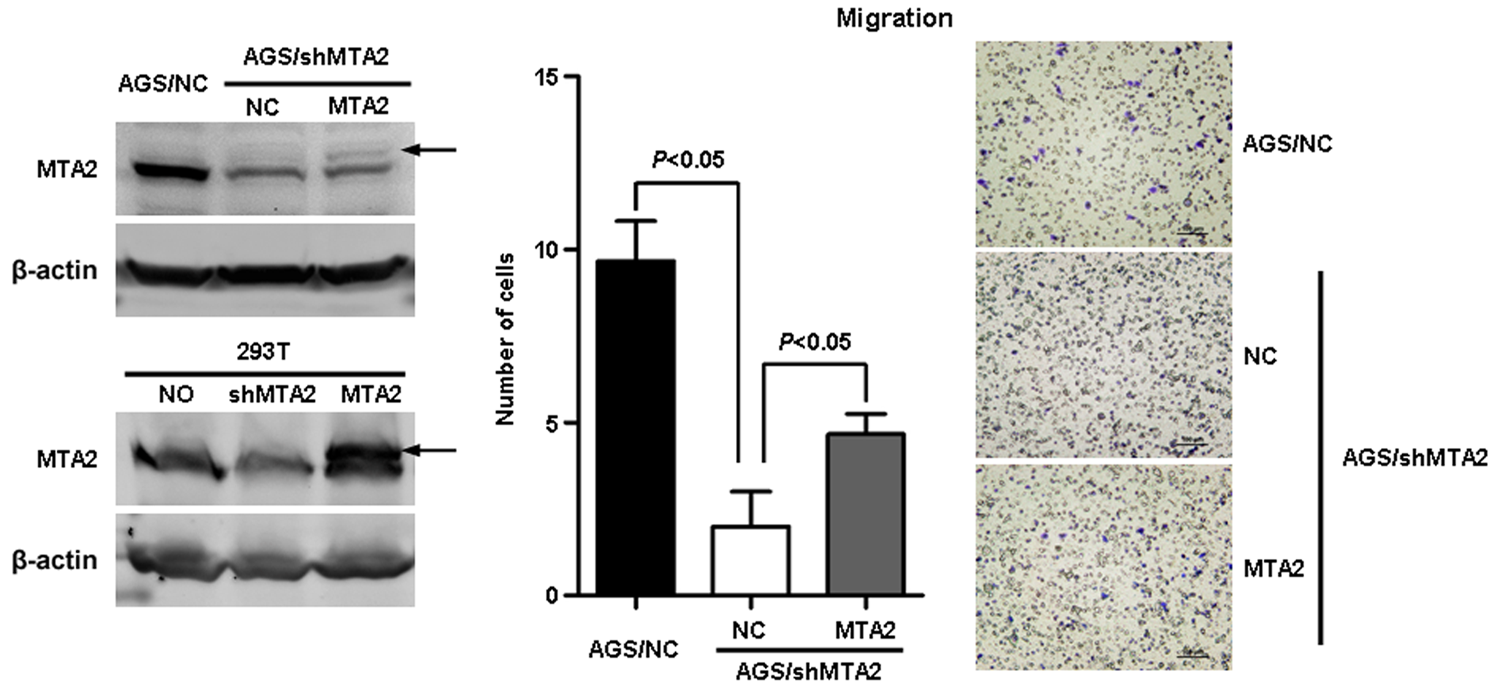


Supplementary Figure S2


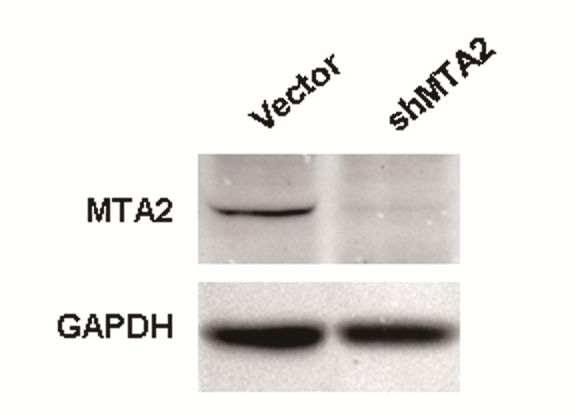


Supplementary Figure S3


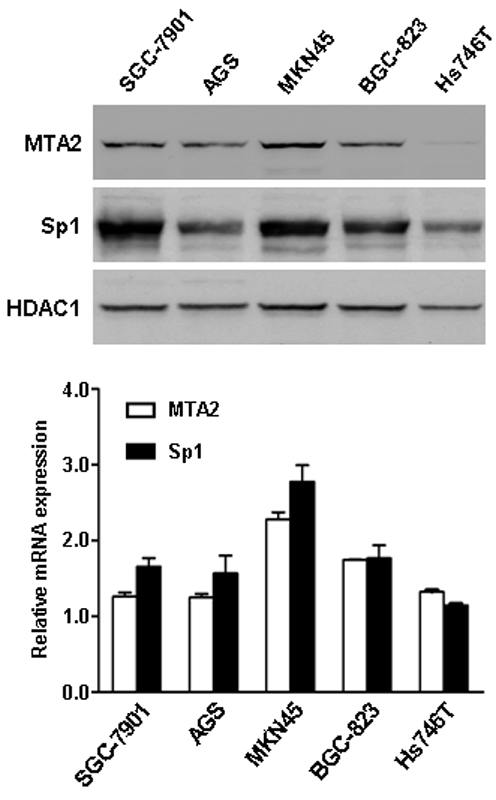


Supplementary Figure S4


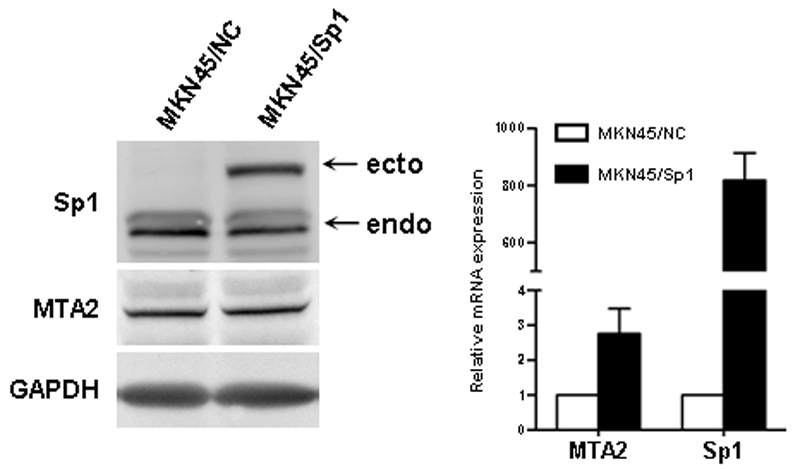


Supplementary Figure S5


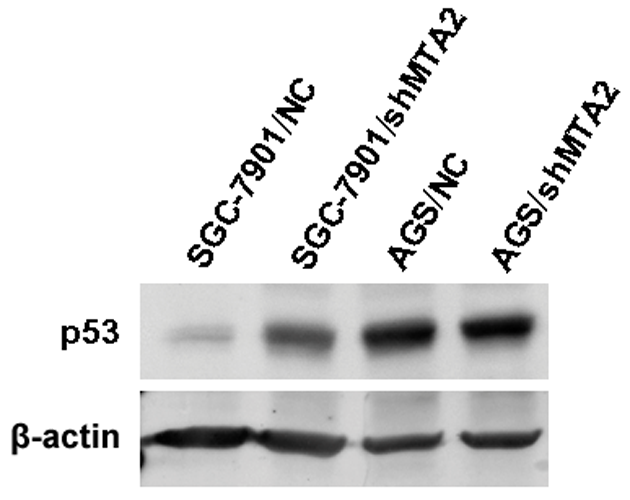


Supplementary Figure S6

Supplementary Table S1 Clinicopathological characteristics of 127 gastric cancer patients

| Clinicopathological characteristics | | N | | % |
| --- | --- | --- | --- | --- |
| Age | | | | |
|  | median | 63 |  | |
|  | Range | 28-84 |  | |
| Gender | | | | |
|  | male | 82 | 64.6 | |
|  | female | 45 | 35.4 | |
| Tumor invasion | | | | |
|  | T1 | 13 | 10.2 | |
|  | T2 | 23 | 18.1 | |
|  | T3 | 75 | 59.1 | |
|  | T4 | 16 | 12.6 | |
| Lymph node metastasis | | | | |
|  | negative | 49 | 38.6 | |
|  | positive | 78 | 61.4 | |
| TNM staging | | | | |
|  | I | 29 | 22.8 | |
|  | II | 27 | 21.3 | |
|  | III | 45 | 35.4 | |
|  | IV | 26 | 20.5 | |
| Lauren’s type | | | | |
|  | Intestinal | 59 | 46.5 | |
|  | diffuse | 68 | 53.5 | |
| Differentiation | | | | |
|  | high | 31 | 24.4 | |
|  | low | 96 | 75.6 | |

Supplementary Table S2 Primer for PCR in chromatin immunoprecipitation

| Primer | | Sequence | Position | Product |
| --- | --- | --- | --- | --- |
| Primer 1 | F | GATTCACGCACGACGACTC | -1179bp | 336bp |
|  | R | CAGCGGGTCTAAACAGTGTG | -843bp |  |
| Primer 2 | F | GACTAGGGGCCGACTCTTTC | -948bp | 275bp |
|  | R | TACGCTCCCTTACCCTTTCC | -671bp |  |
| Primer 3 | F | GTTAAGCCCGACTCCTCTCC | -559bp | 258bp |
|  | R | CGGAGTCCCACTAGTCGTTG | -301bp |  |
| Primer1.1 | F | GATTCACGCACGACGACTC | -1179bp | 156bp |
|  | R | ATCAAACGTCGAGGGAGGAG | -1023bp |  |
| Primer1.2 | F | CTCCTCCCTCGACGTTTGAT | -1043bp | 200bp |
|  | R | CAGCGGGTCTAAACAGTGTG | -843bp |  |

Supplementary Table S3 Primers for construction of luciferase reporter plasmid

| Plasmid | | Sequence | Position |
| --- | --- | --- | --- |
|  | R | GAAGATCTCCCACCCGGTACATGTTGG | 26bp |
| pGL3-117 | F | GGGGTACCCAGTGAGACTCCCTCGAAGC | -117bp |
| pGL3-482 | F | GGGGTACCGACAGGAGCCGAACTTCTTC | -482bp |
| pGL3-797 | F | GGGGTACCTGAGGGGCAATAAACCAGA | -797bp |
| pGL3-1042 | F | GGGGTACCTCCTCCCTCGACGTTTGAT | -1042bp |
| pGL3-1179 | F | GGGGTACCGATTCACGCACGACGACTC | -1179bp |
| pGL3-264 | F | GGGGTACCTCCTCCCTCGACGTTTGAT | -1042bp |
|  | R | GAAGATCTTCTGGTTTATTGCCCCTCAG | -778bp |
